# Epigenetic regulation of transgenes

**DOI:** 10.1016/j.jbiotec.2026.05.015

**Published:** 2026-05-28

**Authors:** Leila Mwangi, Eric Warga, Jacob Elmer

**Affiliations:** Villanova University, Department of Chemical & Biological Engineering,, 800 E Lancaster Avenue, Villanova, PA 19085, USA

**Keywords:** Plasmid DNA, Restrictosome, SUMOylation, Transgenes, PML Nuclear Bodies (NBs), DNA Methylation, Histone Modifications

## Abstract

Gene therapy holds significant potential for treating genetic disorders, but the use of viral vectors is limited by factors such as immunogenicity, payload capacity, and high manufacturing costs. Nonviral gene delivery (NVGD) using plasmid DNA presents an attractive alternative; however, it typically provides a limited magnitude or duration of transgene expression. One potential reason for these shortcomings is the host cell’s epigenetic regulation mechanisms, which can silence both viral and nonviral transgenes. Specifically, when foreign DNA enters the nucleus, it is detected by nuclear DNA sensors, such as IFI16, which initiate the assembly of a “restrictosome” or nuclear domain 10 (ND10) body. This multiprotein complex contains several components, such as PML, Speckled Proteins (e.g., SP100), DAXX, and ATRX that act as a scaffold for recruiting various epigenetic modifiers that subsequently deposit repressive histone modifications like H3K9me3 and H3K27me3 on the transgene chromatin. These marks induce DNA methylation and the subsequent condensation of plasmids or episomes into heterochromatin, which represses transgene expression. Alternatively, unmethylated CpG motifs in bacterial plasmid DNA can trigger innate immune responses in the cytosol, but this review will specifically focus on the detailed mechanisms of epigenetic regulation responsible for silencing plasmid DNA within the host cell nucleus. Addressing these nuclear defense mechanisms, potentially through strategies that manipulate DNA methylation or inhibit restrictosome activity, is crucial for advancing the development of safe, effective, and long-lasting plasmid viral and non-viral gene therapies.

## 1. Introduction

Therapeutic transgenes have been used to enhance, complement, or replace faulty genes that are responsible for a wide variety of genetic disorders. As of 2025, only viral vectors have been used in the clinic for gene delivery due to their relatively high transduction efficiency; but each type of virus has its own unique advantages and disadvantages (Naldini, 2015). For example, lentiviruses (LVs) are commonly used to ensure stable, long-term transgene expression, as they can integrate their genetic cargo into host cell chromosomes in both dividing and quiescent cells. Indeed, lentiviruses have been used for several types of CAR-T cell therapy for various cancers (e.g., Kymriah, Yescarta, Breyanzi, Abecma, Carvykti) (Shchaslyvyi et al., 2023) and a range of genetic disorders (e.g., Skysona for Cerebral Adrenoleukodystrophy (CALD), Zynteglo for β-thalassemia, Libmeldy for Metachromatic Leukodystrophy (MLD)) (Shchaslyvyi et al., 2023). However, lentiviruses have a limited payload capacity (Tailor, 2016) and integrate genes at semi-random locations in the host cell genome, which can lead to the activation of proto-oncogenes (e.g., *LMO2*) or repression of tumor suppressor genes, (Hacein-Bey-Abina et al., 2003) thereby inducing myelodysplastic syndrome (MDS).

Adeno-associated viruses (AAV) are also commonly used for gene therapy, but they deliver genes as episomes that are not intended to integrate into the host cell’s genome (Darrow, 2019). This characteristic makes AAVs inherently safer than LVVs, but it also potentially limits the durability of transgene expression over time in cells and tissues with high turnover rates. Nonetheless, several AAV gene therapies have been approved in the U.S. and the E.U. to treat genetic disorders, including Zolgensma for Spinal Muscular Atrophy (SMA), Hemgenix for Hemophilia B, Upstaza for Aromatic L-Amino acid Decarboxylase (AADC), and Luxturna for Retinal Dystrophy (Shchaslyvyi et al., 2023; Mücke et al., 2024). While some of these therapies have shown sustained expression and therapeutic benefits, some patients receiving AAV gene therapies (particularly those with Hemophilia) have eventually had to return to prophylaxis after the levels of transgene expression decreased over time (Frenzel et al., 2023). It is also worth noting that AAVs have a smaller genetic payload capacity (4.7 kbp) (Wu et al., 2010) than LVs and AAVs may induce hepatotoxicity, since the liver acts as a sink for systemically delivered AAV vectors. AAVs also cannot be readministered because patients’ immune systems develop neutralizing antibodies against the AAV capsid that persist for years (Ronzitti et al., 2020). Finally, the cost of both lentiviral and AAV gene therapies are relatively high, ranging from $0.5 to 4 million USD (Collins et al., 2023).

While lentiviral and AAV gene therapies have certainly saved many lives, their safety concerns and other limitations highlight the necessity for alternative non-viral gene delivery methods that enhance safety without compromising efficacy. Plasmid-based gene delivery represents one such alternative that offers multiple advantages. First of all, plasmids can be generated at a significantly lower cost than viral vectors (Darrow, 2019). Plasmids can also accommodate much larger genetic payloads, although the size of a plasmid tends to be inversely proportional to its transfection efficiency (Ribeiro et al., 2012). Additionally, plasmids can function either as episomes for sustained transgene expression in quiescent cells or as donors for Cas9-mediated genome editing or integration via transposases, thereby providing comparable long-lasting transgene expression profiles to lentiviral vectors (albeit with a relatively lower integration efficiency in some cases) (Kreppel and Hagedorn, 2022). Despite these advantages, only a few plasmid-based non-viral gene therapies have reached the clinic, such as Collategene (Ylä-Herttuala, 2019) (approved in Japan in 2019, but discontinued in 2024) and Neovasculgen (Koteneva et al., 2024) (approved in Russia in 2011). Both of these therapies were designed to treat Critical Limb Ischemia (CLI), but neither the E.U. nor the U.S. have approved its use. Collatagene utilizes plasmid DNA to encode the human hepatocyte growth factor gene for the treatment of CLI and Periphery Artery Disease (PAD), particularly in patients where revascularization is relatively challenging. In the U.S. and E.U., Collatagene completed Phase II and III clinical trials (NCT04267640), showing promise for ulcer healing, pain reduction, and extending the time to amputation or death. However, Collatagene was discontinued in 2024 due to reports of inconsistent overall efficacy outcomes, and the impact on patients’ quality of life remains unclear (Hakariya et al., 2025). There were also reports of the inability to replicate clinical studies results during post-market surveillance (Kawaguchi, 2025).

**Fig. 1.**
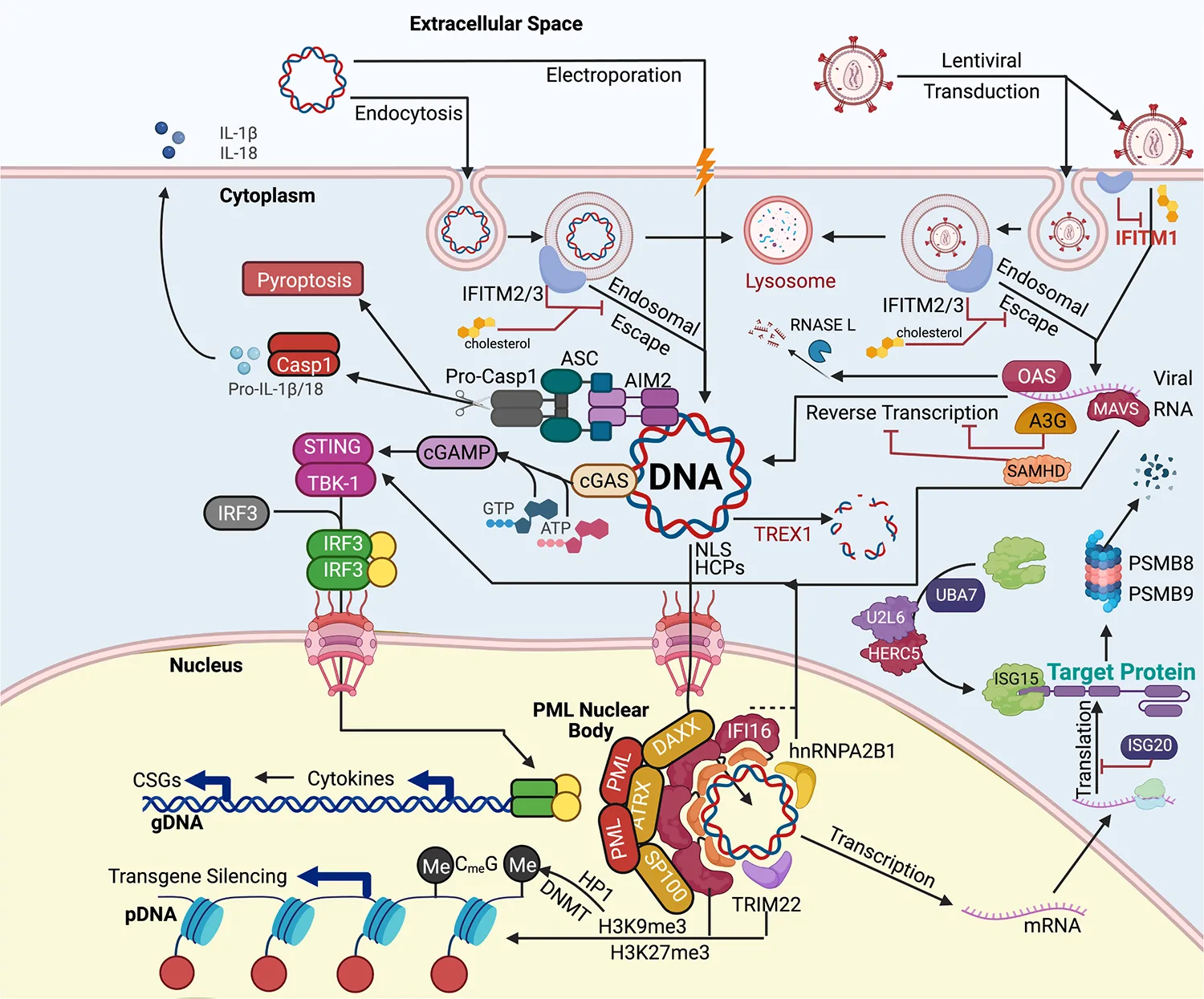
Sequence of events from plasmid/viral entry into the cytoplasm, through sensing, restrictosome assembly, histone modification, DNA methylation, and eventual silencing of the transgene (Bürckstümmer et al., 2009; Zhu et al., 2019; De Miguel et al., 2021; Sutter et al., 2024; Johnson et al., 2014; Ma et al., 2022; Mitchell et al., 2014; Gulve et al., 2022; Clatterbuck Soper et al., 2024; Xu et al., 2016; Liu et al., 2018; Almine et al., 2017; Unterholzner et al., 2010; Stratmann et al., 2015; Lum et al., 2019; Fada et al., 2021; Zimmerman et al., 2018) (Figure created on BioRender.com).

Overall, NVGD has faced significant challenges in effectively delivering and expressing transgenes, despite offering a high degree of safety and customization. Significant progress has been made in developing more efficient delivery methods for these vectors, including chemical approaches such as cationic lipids and physical techniques like electroporation and sonoporation (reviewed elsewhere) (Al-Dosari and Gao, 2009; Zu and Gao, 2021; Zhang and Khan, 2024; Harris and Elmer, 2021; Krut et al., 2022). However, regardless of how a gene is delivered into a cell, it will eventually encounter host cell defense proteins and epigenetic regulatory mechanisms that repress the expression of foreign genes (as shown in Fig. 1 and listed in Table 1). Indeed, the importance of epigenetic repression of transgenes has been highlighted by several studies, which have shown that while episomes can be detected in cells for several months after transfection, transgene expression decreases after a few days or weeks (Ochiai et al., 2007; Brooks et al., 2004; Yang et al., 2010; Gracey Maniar et al., 2012; Das et al., 2022; Roy and Ghosh, 2024).

In contrast, most viruses are able to evade these host cell defenses and sustain high level expression by expressing proteins that overcome the cell’s natural barriers and maintain expression of their genes (Li, 2023)). For example, the Herpes Simplex Virus 1 (HSV−1) protein ICP0 (Infected Cell Protein 0) counteracts host cell immune defenses by triggering the degradation of key components of the innate immune response, such as IFI16 (Interferon Gamma Inducible Protein 16), PML (Promyelocytic Leukemia) and other ND10 (Nuclear Domain–10) body-associated proteins (Knipe, 2015; Mallon et al., 2012). Specifically, ICP0 has E3 ubiquitin ligase activity, which allows it to mark these proteins for proteolysis, thereby preventing epigenetic silencing of viral genes (Le Hars et al., 2024)). Alternatively, the IE1 (Immediate Early Protein 1) protein of human cytomegalovirus (hCMV) prevents inflammation by modulating the JAK-STAT signaling pathway. It also accumulates in the host cell nucleus, where its C-terminal domain contains motifs for SUMOylation (small ubiquitin-like modifier) and chromatin tethering. These features allow IE1 to bind SUMO1 and the H2A-H2B acidic pocket on nucleosomes(Harwardt, 2016). While the IE1-SIM1(SUMO-interacting motif) interaction has been shown not to play a role in inhibiting the SUMOylation of other antiviral proteins (e.g., PML)(Schilling, 2016), it may facilitate interaction with SUMOylated transcription factors such as TAF12 or co-regulators to enhance trans-activation of viral genes through its interaction with SIM2, which then increases recruitment of Histone Acetyltransferases (HATs) and RNA Polymerase II,to create a permissive environment for gene expression (Tripathi et al., 2021). These examples highlight the importance of considering host cell epigenetic regulatory mechanisms in the design of non-viral gene therapies that typically lack specialized proteins to facilitate cellular entry, nuclear import of the transgene, and maintenance of transgene expression (Colella et al., 2017).

While several reviews have summarized the wide range of antiviral host cell defenses (Coroadinha, 2023; Annoni et al., 2019; Follenzi et al., 2000; Muhuri et al., 2021; Prasad et al., 2022; Mano et al., 2015; Colella et al., 2017), and cytosolic DNA sensing pathways (Zahid et al., 2020; Won and Bakhoum, 2020; Zheng, 2020; Chen et al., 2016; Briard et al., 2020; Yu and Liu, 2021) in eukaryotic cells, this review will focus on the epigenetic regulation of both viral genes and plasmid DNA in the host cell nucleus, with an emphasis on DNA methylation, post-translational modifications to histones, binding by non-coding RNAs, and chromatin remodeling (Hamilton, 2011). This paper will also review the nuclear DNA sensing pathways and internal cellular mechanisms that can repress NVGD. Strategies to inhibit these regulatory mechanisms and enhance transgene expression will also be discussed, if available.

## 2. Cytosolic and nuclear DNA sensing

Regardless of which nonviral vector is used for non-viral gene delivery, a common outcome is the release of plasmid DNA into the cytosol of the host cell. In contrast, most viral vectors use tegument proteins to traffic their cargo directly into the nucleus. This causes significant challenges for non-viral vectors as the presence of foreign DNA in the cytosol triggers the innate immune response.

Several cytosolic DNA-sensing pathways work to detect foreign DNA in the cytosol, including the TLR9/MyD88 (Toll-Like Receptor 9), cGAS/STING (Stimulator of Interferon Genes), and AIM2 (Absent in Melanoma 2) pathways. For example, AIM2 is a member of the PYHIN (Pyrin and HIN domain) protein family that is upregulated by interferon β and specifically binds to double-stranded DNA in the cytosol (Bürckstümmer et al., 2009). This interaction induces a conformational change that triggers the assembly of an inflammasome, which includes other proteins such as Caspase–1 and IL–18 (interleukin 18) that facilitate pyroptosis (Zhu et al., 2019; De Miguel et al., 2021).

In addition to well-established cytosolic DNA sensing pathways (e.g., cGAS/STING), many cell types also have nuclear DNA sensors that can differentiate between host cell DNA and foreign DNA. Overall, these nuclear DNA sensing pathways involve an initial binding event between foreign DNA and a nuclear protein (e.g., IFI16), which then triggers the formation of larger complexes with additional proteins (e.g., PML, ATRX, DAXX, and Sp100) that can either directly block transcription or induce modifications to the DNA or associated histone proteins that indirectly silence transgenes. A summary of studies that investigated the roles of known nuclear viral restriction factors via Cas9 knockout is shown in Table 2.

### 2.1. IFI16

**Fig. 2.**
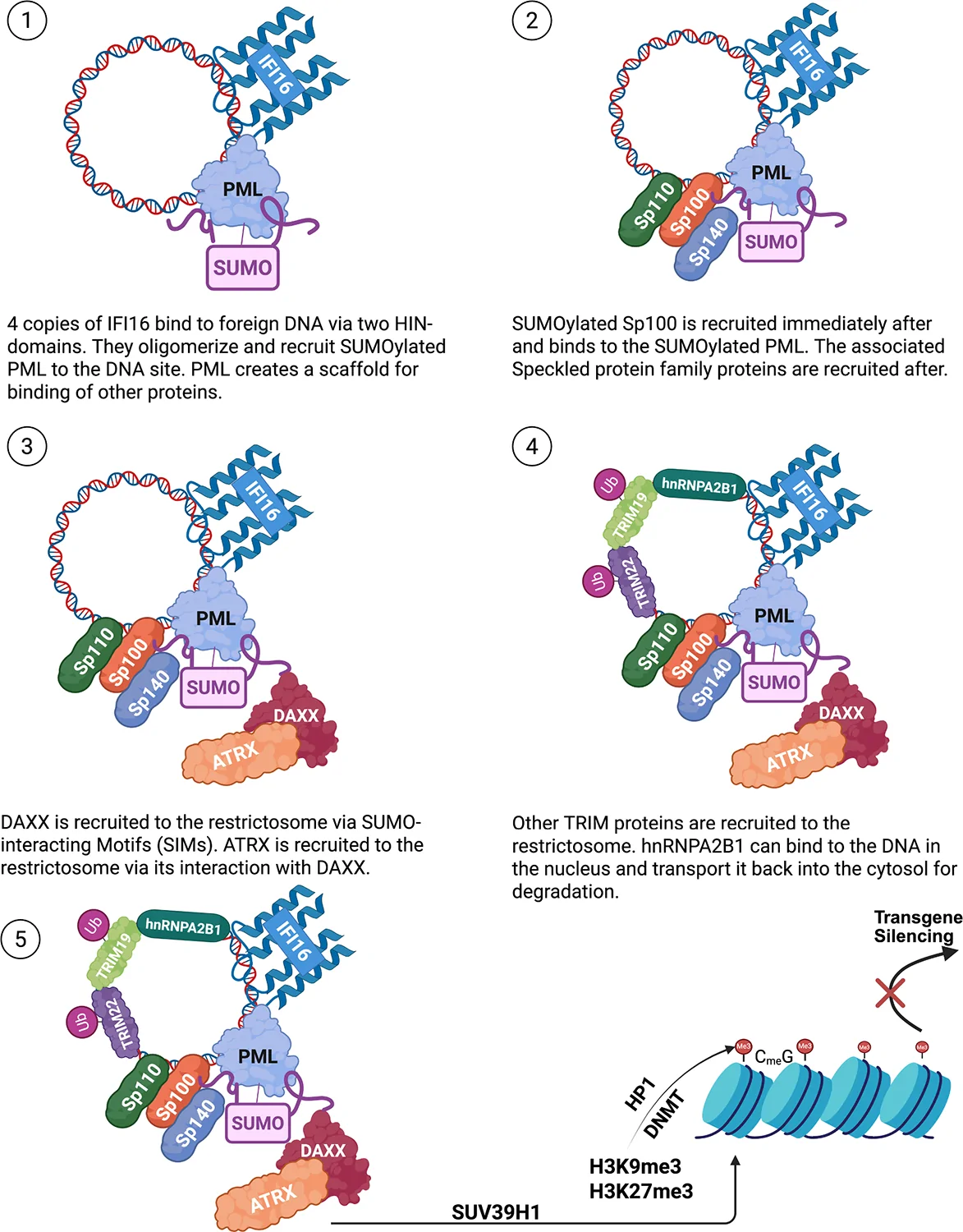
Formation of the restrictosome and its impact on plasmid DNA. The formation of the restrictosome starts with four copies of IFI16 binding to the plasmid. PML then undergoes SUMOylation through a SUMO binding motif. Sp100 and other speckled proteins are then recruited in a SUMO-dependent fashion. DAXX is also recruited to the restrictosome via SUMO-interacting motifs (SIMs), and ATRX is recruited to the restrictosome through its association with DAXX. The assembly of the restrictosome leads to transgene silencing and the repression of gene transcription. (Figure created on BioRender.com).

Like AIM2, IFI16 is another PYHIN protein that can activate the innate immune response in the cytosol through the cGAS-STING pathway to upregulate interferons, cytokines, and cytokine-stimulated genes (CSGs) (Won and Bakhoum, 2020; Zheng, 2020; Chen et al., 2016; Briard et al., 2020; Almine et al., 2017). IFI16 has also been shown to non-specifically bind to DNA in the nucleus through its two HIN–200 domains (Unterholzner et al., 2010). These HIN domains may bind either genomic or foreign DNA in the nucleus, but a distinguishing characteristic of foreign DNA is that it lacks the histones and proteins that saturate genomic DNA. Consequently, as foreign DNA enters the nucleus, it can be bound by several copies of IFI16. If at least 4 copies of IFI16 bind the foreign DNA, the pyrin domains of IFI16 oligomerize and trigger the formation of a “restrictosome” (Figs. 1 and 2).

The restrictosome is a large protein complex that recruits additional epigenetic factors (e.g., PML, ATRX, and DAXX) to foreign DNA in the nucleus to facilitate specific repressive modifications on the histones bound to transgenes (e.g., H3K9me2/3 and H3K27me3) or directly methylate the DNA (Fada et al., 2021; Li et al., 2021; Lum et al., 2019; Merkl and Knipe, 2019; Roy and Ghosh, 2024; Stratmann et al., 2015; Suzuki, 2016; Weinberg, 2019). Indeed, our group and others have detected these repressive histone modifications and DNA methylation on plasmids and viral episomes across multiple cell types (Brooks, 2004; Johnson, 2014; Merkl and Knipe, 2019; Ngai et al., 2015; Rajeevkumar et al., 2015; Roy et al., 2019; Zimmerman et al., 2018), including the H3K9me3 and H3K27me3 modifications that are commonly associated with heterochromatin and silenced genes (Günther et al., 2014).

In addition to recruiting epigenetic modifiers, IFI16 can also interfere with transgene expression independently of other restrictosome proteins by directly blocking RNA polymerase and transcription factors from binding to promoters (e.g., CMV) (Brzostek-Racine et al., 2011; Orzalli et al., 2013; Sutter, 2024; Tan, 10AD). Indeed, overexpression of IFI16 in HEK-293T decreases transgene expression compared to wild-type cells, even though those cells lack other key DNA sensing proteins (e.g., STING and TBK–1) (Mitchell et al., 2014; Warga et al., 2022). Furthermore, Sutter et al. demonstrated that knocking down IFI16 with siRNAs in human fibroblasts increased AAV transgene expression, which was transcriptionally silenced in control cells that were not treated with siRNAs(Sutter et al., 2024).

### 2.2. PML and other TRIM (Tripartite Motif-Containing) proteins

Following the initial detection of foreign DNA in the nucleus by IFI16, one of the first proteins subsequently recruited to this complex is the Promyelocytic Leukemia protein (PML), which plays a central role in epigenetic silencing. PML, also known as TRIM19 (tripartite motif-containing protein 19), has been documented to interact directly or indirectly with more than 160 proteins. It is important for the assembly of ND10 bodies, which have been shown to silence bound genes. In general, TRIM (tripartite motif-containing) proteins, like PML, feature an N-terminal tripartite RBCC motif (i.e., RING (Really Interesting New Gene), B-box1/2, coiled-coil) and exhibit various structural variations in the C-terminal region (Tan, 10AD). The RING domains in several TRIM proteins have E3 ubiquitin ligase activity, enabling the proteins to catalyze ubiquitylation, SUMOylation, or ISGylation (i.e., conjugation of interferon (IFN)-stimulated gene 15) of themselves or other target proteins (e.g., DAXX and ATRX) (Corpet, 2020; Patra and Müller, 2021). The CC domain facilitates protein oligomerization, which can lead to the assembly of protein complexes in the nucleus or cytoplasm.

PML recruitment to ND10 bodies directly affects transgene silencing and decreases viral replication. Mitchell et al. examined the impact of knocking out PML on AAV transduction using PML knockout mice. They found that eliminating PML can increase the transduction efficiency of recombinant AAV serotype 2 (rAAV2) by up to 28-fold in female mice, directly implicating PML in the silencing of AAV transgenes (Mitchell et al., 2014).

PML utilizes its RING-box-coiled domain to oligomerize and provide a scaffold for the binding of the other ND10 proteins. It also contains a SUMO (small ubiquitin-like modifier)-binding motif that enables direct, non-covalent interaction within nuclear bodies (NB). Both this motif and the RING-Box domain are crucial for forming PML-NBs (Shen et al., 2006; Tan, 10AD). The RING tetramer recruits the SUMO E2 conjugating enzyme, Ubc9. Additionally, PML facilitates the recruitment of other ND10 body proteins through SUMOylation-dependent mechanisms (Shen et al., 2006). For example, the recruitment of Sp100 and the associated Speckled protein family (SP110, SP140, and SP140L) is SUMO-dependent (Chen et al., 2008; Dellaire et al., 2006). DAXX also has SUMO-interacting motifs (SIMs) that facilitate binding to SUMOylated PML (Corpet, 2020; Dellaire et al., 2006). ATRX depends on its interaction with DAXX and therefore follows DAXX in forming the restrictosome (Corpet, 2020; Tang, 2004).

PML may also silence transgenes by repressing transcription through interactions with histone deacetylase (HDAC) (Wu et al., 2001). Wu et al. demonstrated that PML regulates histone deacetylation by recruiting HDAC to transgene promoters (Wu et al., 2001)(). They initially overexpressed PML in several cell lines (e.g., U2OS) by infecting them with a recombinant PML adenovirus (Ad-PML). PML was then immunoprecipitated from the cells with a polyclonal antibody to show that PML was bound to HDAC1. Furthermore, when U2OS cells were transfected in the presence of the HDAC inhibitor trichostatin A (TSA), an increase in transgene (luciferase) activity was observed (Wu et al., 2001).

After PML is recruited, it serves as a structural scaffold for the restrictosome complex. The RING domains within PML, along with other TRIM family proteins, have E3 ubiquitin ligase activity that allows these proteins to facilitate the processes that are critical for regulating protein function and stability such as ubiquitylation, SUMOylation, or ISGylation. These processes can involve modifying their own structure or adding ubiquitin, SUMO, or ISG constructs to other target proteins such as DAXX and ATRX, (), thereby influencing various cellular pathways and responses.

### 2.3. Ubiquitinylation and SUMOylation

As previously mentioned, two common post-translational modifications (PTMs) observed on PML, histones, and other restrictosome proteins are SUMOylation and ubiquitinylation. In general, SUMO and ubiquitin conjugation regulate cellular functions like protein stability, transport, cell division, and the innate immune response. Ubiquitin, a 76 amino acid protein, can be conjugated to proteins as a single subunit or as a chain of multiple subunits (Guo et al., 2023; Lee et al., 2023). For example, monoubiquitinylation of histone H2B relaxes chromatin, facilitating transcription, and modulating gene expression (Zhu et al., 2005; Farrona and Barneche, 2025), while monoubiquitinylation of histone H2A is linked to transcriptional repression (Farrona and Barneche, 2025; Zhou et al., 2008; Barbour et al., 2020). Ubiquitinylated histones also recruit chromatin remodelers (e.g. RSF1 (Zhang et al., 2017)) and transcription factors (e.g. FOXO1 (Lee et al., 2023)) which could either activate or repress transcription.

Like ubiquitinylation, SUMOylation is a process that involves the conjugation of the small ubiquitin-like modifier (SUMO) protein to target proteins, thereby affecting their localization, transcription, and interactions. Human SUMO isoforms include SUMO–1, SUMO–2, SUMO–3, and SUMO–4 (Brackett and Blagg, 2021). SUMOylation is regulated by an enzyme cascade that involves with an ATP-dependent heterodimeric SUMO-activating E1 enzyme, a SUMO conjugating E2 enzyme, and SUMO E3 ligases that help bind the substrates that are subsequently SUMOylated. SUMOylation is also a reversible process that can be undone by SUMO/sentrin-specific peptidases (SENPs) that hydrolyze SUMO from target proteins (Brackett and Blagg, 2021).

Ubc9 is the sole E2 enzyme for SUMOylation in humans, while several different E3 proteins like PML confer substrate specificity and enhance conjugation efficiency (van Damme et al., 2010; Tahmasebi et al., 2014). Shastrula et al. showed that the knockdown of Ubc9 in HeLa cells prevented recruitment of DAXX at PML nuclear bodies. They also showed that PML and SUMO rings do not form when Ubc9 is depleted, indicating that SUMOylation is required for these processes (Shastrula et al., 2019). Shogren-Knaak et al. also studied how SUMOylation targets PML and SUMO to transgenes by knocking down Ubc9, PML, DAXX, and ATRX with shRNAs in HeLa cells. H3K9me3 levels around the transgene decreased after DAXX, PML, and Ubc9 depletion, indicating that PML-NBs play a role in regulating heterochromatin formation (Shogren-Knaak et al., 2006). These studies imply that SUMOylation of ATRX and DAXX by Ubc9 represses transgene expression by modifying chromatin structure, which influences DNA accessibility and gene transcription (Shastrula et al., 2019).

### 2.4. Speckled protein family

When PML undergoes post-translational modifications such as conjugation of SUMO molecules, it transforms into a scaffold structure which plays a crucial role in the formation of the restrictosome. This modified PML then recruits various helper proteins, including Sp100, which belongs to the speckled protein family known for its involvement in nuclear body formation and transcriptional regulation. Sp100 and related speckled proteins (Sp110, Sp140, and Sp140-L) function as chromatin readers that connect unmethylated CpG-rich DNA to repressive chromatin states. Speckled proteins (SPs) exhibit a distinctive localized pattern within the nucleus, forming irregular or “speckled” patterns when visualized with immunofluorescence microscopy (Spector and Lamond, 2011; Fei et al., 2017). These proteins have a nuclear localization signal (NLS) and additional functional domains that allow them to function as “chromatin readers” (Fraschilla and Jeffrey, 2020). Chromatin readers serve as key interpreters of the epigenome that facilitate cell-specific transcriptional programs. For example, SPs possess a plant homeodomain (PHD) that can recognize histone methylation, as well as a bromodomain (BRD) that binds acetylated lysines on histones (Fraschilla and Jeffrey, 2020). Some SPs also feature a SAND domain (Sp100, AIRE–1, NucP41/75, and DEAF–1) that directly binds unmethylated CpGs that are present in bacterial plasmid DNA through a positively charged surface region with a KWDK motif that may enhance protein-protein interactions with ND10 nuclear bodies (Bottomley et al., 2001). Therefore, Sp100 and Sp110 may bind foreign DNA directly or via interactions with PML and associated histones. Many cell types express Sp100 and Sp110, but Sp140 is found exclusively in immune cells. Specifically, RNA-seq data demonstrate that all SPs are highly expressed in developing and mature B cells, as well as the activated CD4^+^ and CD8^+^ T cells used in CAR-T cell therapy. SPs are also interferon (IFN)-stimulated genes (ISGs) that are upregulated during the innate immune response to pathogen-associated molecular patterns (PAMPs) like plasmid DNA (Fraschilla and Jeffrey, 2020).

The Sp100 gene encodes 11 protein-coding isoforms, four of which have been extensively studied (A, B, C, and HMG). Each of these isoforms has a common N-terminal region with an NLS, a destruction-box (D-box) that is necessary for proteasomal degradation, sequences essential for Sp100 dimerization and PML-NB localization, two SUMO-conjugating motifs (SCM), a SUMO-interacting motif (SIM), and a trans-activating region. While the common N-terminus of Sp100 mainly facilitates protein-protein interactions, the C-termini of each isoform determines its interactions with DNA and histones (see Fig. 2). For example, Sp100B, C, and HMG possess high mobility group (HMG) and SAND domains that facilitate specific interactions with unmethylated CpG motifs on viral or plasmid DNA. The SIM domain of Sp100 allows it to interact with the heterochromatin protein 1 alpha (HP1-α) which binds H3K9 marks and condenses DNA into heterochromatin (Nakamura et al., 2024). However, whether Sp100 directly influences transgene transcription or operates through its interactions with host DNA or chromatin remains unclear(Collados Rodríguez, 2020).

In human macrophages, Sp140 mainly inhibits exogenous gene expression by binding selectively to heterochromatin regions. Recent studies show that after docking on repressive chromatin, typically identified by high levels of H3K27me3 and low levels of H3K4me3, SP140 recruits and interacts with chromatin-remodeling and DNA unwinding proteins such as topoisomerase 1 (TOP1). Sp140 negatively regulates topoisomerase activity, preventing underlying chromatin from unwinding and thereby inhibiting transcription (Amatullah, 2022; Fraschilla and Jeffrey, 2020; Tamburri, 2024). Cable et.al investigated the mechanism by which Sp140L restricts EBV (Epstein Barr virus) gene expression. Their work showed that the EBV latency protein (EBNA-LP) enhances viral replication by inhibiting the speckled proteins Sp100 and Sp140L, while viral replication decreased in cells infected with EBNA-LP deficient (LPKO) viruses. However, Cas9 knockout of the Sp140L gene in B cells allowed the LPKO viruses to replicate, indicating that Sp140L can act as a viral restriction factor(Cable et al., 2025).

### 2.5. Histone modification by DAXX and ATRX

Sp100 contains a DNA-binding domain that can recognize acetylated and methylated histones. This allows it to respond to the condition of the histones bound to DNA and recruit factors that can either compact or relax the chromatin through histone modifications. DAXX and ATRX are examples of such factors that are recruited after Sp100 and play a role in regulating the chromatin.

DAXX and ATRX are important components of the restrictosome that are recruited following the SUMOylation of PML and Sp100 (Negorev et al., 2006). They can recruit several additional proteins, including histone variants, histone modifiers, transcription factors, and chromatin remodelers (Fada et al., 2021). For example, DAXX and ATRX promote chromatin remodeling by facilitating the deposition of histone variant H3.3 into local nucleosomes, (Goldberg, 2010) which subsequently leads to heterochromatin formation (Elsässer, 2012; Lee et al., 2023; Shastrula, 2019). DAXX and ATRX also play a role in targeting heterochromatic regions, inhibiting demethylase activity, and promoting the deposition of additional repressive chromatin marks (Choi et al., 2024).

Hoelper et al. demonstrated that shRNA-mediated knockdown of the DAXX-ATRX complex in murine embryonic stem cells (mESCs) resulted in a 4-fold upregulation of intracisternal (*IAP*) and *Mus musculus* type D (*MusD*) elements (Hoelper et al., 2017). *IAP* and *MusD* are endogenous retroviral transposons in the mouse genome which are classic targets for studying how mammalian cells repress retroviral DNA through histone variant deposition and methylation. This observation suggests that the DAXX-ATRX complex may also be involved in the repression of exogenous transgenes (O’Riordan et al., 2008; Hoelper et al., 2017).

In addition to H3.3, DAXX recruits several epigenetic modifiers like EZH2, a histone methyltransferase that represses genes by adding H3K27me3 marks to nearby histones (Dyer et al., 2017). HP1 then binds histones with H3K9me2/3 and recruits histone methyl transferases to spread the methylation mark to newly bound histone proteins. Finally, the formation of a complete restrictosome with ATRX and DAXX leads to the recruitment of several other epigenetic repressors that make repressive marks to the transgene or associated histones, including SUV39H1, HDAC1, LSD1, UBTF, PAF1, and GLP (Bottomley, 2001; Vaute et al., 2002).

### 2.6. hnRNPA2B1

If foreign DNA enters the nucleus without being detected by the restrictosome proteins, it can still be detected by other proteins, like the nuclear protein hnRNPA2B1. This protein acts a nuclear DNA sensor that transports foreign DNA back into the cytosol, where it must contend with DNA sensors and nucleases again.

The hnRNPA2B1 protein is a heterogeneous nuclear ribonucleoprotein (hnRNP) composed of both an RNA-binding protein component and associated RNA molecules that form ribonucleoprotein particles. The protein component of hnRNPA2B1 consists of RNA recognition motifs (RRMs) and a glycine-rich domain that facilitates dimerization and interaction with other proteins. The RRM domain also allows hnRNPA2B1 to bind host cell mRNAs and enhance their processing, splicing, or nuclear export (Maceratessi and Sampaio, 2024; Zhang et al., 2019).

In addition to mRNA, hnRNPA2B1 can also bind DNA and induce its export from the nucleus to the cytosol. This relocation not only prevents the transcription of foreign DNA but also makes it susceptible to degradation by nucleases in the cytosol. This process begins when hnRNPA2B1 dimerizes on foreign DNA and then interacts with the arginine demethylase JMJD6 (Wang et al., 2019). JMJD6 demethylates hnRNPA2B1 which subsequently induces its nucleocytoplasmic trans-location. This mechanism has been shown to affect both viral (e.g., HSV–1) and non-viral transgenes (Wang et al., 2019).

In summary, foreign DNA is vulnerable to various innate immune responses. These responses include cytosolic defense mechanisms, intracellular trafficking, and nuclear defense mechanisms. If a transgene successfully reaches the nucleus, it may encounter and be repressed by nuclear proteins such as IFI16. When four copies of IFI16 bind to the foreign DNA strand, they cluster together through pyrin domains and recruit PML, which undergoes SUMOylation. This process creates a scaffold that helps recruit additional helper proteins such as the speckled family proteins, ATRX and DAXX, ultimately forming a complex known as the restrictosome. Additionally, the foreign DNA may encounter hnRNPA2B1, which can transport it back out of the nucleus into the cytosol, where it could encounter all the cytosolic DNA sensors such as AIM2. Alongside these nuclear and cytosolic proteins, foreign DNA is also vulnerable to epigenetic silencing by histones.

## 3. Histone modifications associated with transgene chromatin

In addition to IFI16, core histones (H2A, H2B, H3, H4) also rapidly bind plasmid DNA after it enters the host cell nucleus (Riu et al., 2007). These histones then undergo several post-translational modifications, including acetylation, methylation, phosphorylation, and ubiquitinylation (see Table 3). For example, while newly incorporated histones are predominantly acetylated at sites like H3K9 to enable transcription, methylation marks such as H3K9me2 and H3K9me3 can induce the formation of heterochromatin and transcriptional repression (Upadhyay and Cheng, 2011). Additional heterochromatin-associated proteins like HDAC2, HP1α, and SUV39H1 subsequently accumulate and permanently silence genes while euchromatin-associated histone modifications like H3K4ac decrease (Riu et al., 2007). Therefore, understanding the role of chromatin remodeling is crucial for enhancing transgene expression. Several studies have shown that epigenetic processes can regulate transgenes that are integrated into the genome (e.g., from lentiviral or transposase activity) (Alhaji et al., 2019). Furthermore, episomal transgenes in plasmids or AAV genomes can also be negatively influenced by epigenetic regulatory mechanisms (Alhaji et al., 2019). This review will explore the various types of histone modifications and their significant roles in transgene silencing. Understanding the relationships between these modifications and transcriptional regulation may then be used to regulate or manipulate transgene expression levels.

### 3.1. Histone acetylation

Acetylation of histones regulates gene activity by decreasing the affinity of histones for DNA and increasing chromatin accessibility, thereby making DNA available for proteins to activate gene transcription. Histone acetyltransferase enzymes (HATs) add acetyl groups to histone tails, neutralizing lysine charges, causing hyperacetylation, chromatin relaxation, and promoting euchromatin formation for transcription (Shahbazian and Grunstein, 2007; Butterfield et al., 2024). Conversely, histone deacetylases (HDACs) remove these groups, leading to hypoacetylation, chromatin compaction, and gene repression (Shahbazian and Grunstein, 2007; Eberharter and Becker, 2002). Histone deacetylase inhibitors (HDACi) block HDAC activity, increasing acetylation and opening chromatin, which has been shown to enhance transgene expression (Kim et al., 2013).

### 3.2. Histone methylation

Although histone acetylation is associated with chromatin relaxation and enhanced transcription, histone methylation at specific residues can establish a repressive chromatin environment. Histone methylation at sites such as H3K9, H3K27, and H4K20 involves the addition of methyl groups to lysine or arginine residues by histone methyltransferases (HMTs) (Husmann and Gozani, 2019; Di Lorenzo and Bedford, 2010). This modification plays a pivotal role in shaping chromatin structure, often resulting in chromatin compaction and transgene repression. The formation of condensed heterochromatin is further reinforced by the recruitment of heterochromatin-associated proteins (e.g., HP1) to the methylated histone tails, which help to condense DNA into heterochromatin (Endicott et al., 2022).

H3K9 is a key residue involved in regulating chromatin structure and gene expression, serving as a central point in epigenetic control mechanisms. Acetylation of H3K9 is typically associated with euchromatin, while methylation of H3K9 results in heterochromatin formation and transcriptional repression (Qiang et al., 2011; Karmodiya et al., 2012; Padeken et al., 2022). These modifications are dynamically regulated by specific enzymes, including histone acetyltransferases (HATs), (Shahbazian and Grunstein, 2007) histone deacetylases (HDACs), (Shahbazian and Grunstein, 2007; Eberharter and Becker, 2002) histone methyltransferases (HMTs), and demethylases, (Kaouass et al., 2006) illustrating the complexity and precision of epigenetic gene regulation.

In contrast, methylation at other lysine residues, such as H3K4, H3K48, and H3K79, promotes gene expression (Dong and Weng, 2013). These modifications create a chromatin environment that is conducive to transcriptional activation, thereby facilitating access to transcriptional machinery. Notably, H3K4me3 promotes gene expression and plays a crucial role in guiding and maintaining standard gene expression patterns throughout the lifespan of mammals (Howe et al., 2017). H3K4me3 is bound by TAF, a subunit of TFIID complex, via its PHD finger to promote preinitiation complex assembly and transcription initiation. It facilitates the recruitment and release of RNA polymerase II (RNAPII) from the preinitiation complex, thereby enhancing transcription (Wang et al., 2023; Lauberth et al., 2013).

In addition to H3K9me3, several other histone modifications have been observed in transfected pDNA, as summarized in Table 3 (Ramazi et al., 2020). Histone tail methylation exhibits a dual role in gene regulation, with different methylation patterns either silencing or activating gene expression, highlighting the complexity and versatility of epigenetic regulation in controlling cellular processes (Rose and Klose, 2014).

### 3.3. Histonefection

Beyond their impact on transcriptional regulation, histones also play a practical role in gene delivery because of their strong affinity for DNA. This allows them to package nucleic acids into compact structures. This dual ability to organize DNA and influence gene expression has inspired multiple groups to investigate histones as potential delivery vehicles for transgenes.

The ability of histones to simultaneously bind genes and regulate their expression has inspired multiple groups to use them as gene delivery vehicles (Kaouass et al., 2006). Indeed, histones have several positively charged residues, including lysine and arginine, that promote strong electrostatic interactions with the negatively charged DNA backbone. This enables them to condense DNA, which is important for packaging large plasmids into particles that are effectively taken up by host cells. Studies by Kaouass et al. have shown that histones are effective gene delivery vehicles due to their DNA-binding domains and natural ability to localize in the nucleus via their endogenous NLS (Kaouass et al., 2006).

Killian et al. assembled plasmid DNA and core histones into chromatin using a salt gradient dialysis method. To facilitate targeted delivery, they linked these complexes to bispecific antibody derivatives (bsAbs) via a nucleic acid binding peptide bridge. When these complexes were introduced into MCF-7 cells and GFP-positive cells were analyzed via flow cytometry after 48 h, it was determined that their complexes resulted in 90% GFP^+^ cells, compared to cells transfected with Lipofectamine (60% GFP^+^ cells) (Killian et al., 2019).

Although histones can improve plasmid condensation and facilitate nuclear delivery, efficient entry into the cell does not guarantee sustained transgene expression. Once inside the nucleus, transgenes are still subject to epigenetic regulation, including DNA methylation, which can influence whether a transgene is active or silenced.

## 4. DNA methylation

While previous sections have focused on the role of protein binding and post-translational amino acid modifications in regulating gene expression, it is equally important to consider direct modifications to the DNA itself. The most well-characterized and significant DNA modification is methylation of CpG motifs in genomic DNA. Indeed, this covalent and inheritable modification plays a central role in the epigenetic regulation of both endogenous and foreign genes.

DNA methylation is typically associated with inactive heterochromatin and gene silencing (Szyf, 2003). DNA methylation is primarily mediated by DNA methyltransferases (DNMTs), including Dnmt1, Dnmt3a, Dnmt3b. Dnmt1 maintains existing methylation patterns during replication, while Dnmt3a and Dnmt3b establish *de novo* methylation at CpG sites (Chédin et al., 2002). Transcription factors such as Myc can induce methylation by recruiting these DNMTs to specific loci (Brenner et al., 2005). In mammalian cells, DNMTs add a methyl group to the 5-carbon of cytosine residues within CpG dinucleotides, producing 5-methylcytosine (5mC). CpG islands are stretches of DNA rich in 5mC that serve important roles in gene regulation (Moore et al., 2013; Walsh and Bestor, 1999). In contrast, bacterial DNA methylation primarily occurs at adenine residues (e.g., GA_me_TC) and different cytosine motifs (e.g., CC_me_WGG) (Casadesús and Low, 2006). These differences in methylation patterns are important to mention because plasmids used for gene delivery are typically produced in bacteria, so they are methylated at GATC and CCWGG motifs, but not at CpG motifs (Howe et al., 2017; Walsh and Bestor, 1999; Wang, 2023;. While the absence of CpG methylation may seem beneficial, unmethylated CpG motifs can activate Toll-like receptor 9 (TLR9) during endocytosis, triggering an innate immune response and expression of cytokines that hinder gene delivery (Hyde et al., 2008; Schwartz et al., 1997). However, TLR9 is mainly found in dendritic cells and is absent in most other cells (e.g., T cells) (Alhaji et al., 2019; Chédin et al., 2002; Moore et al., 2013; Lodoen and Lanier, 2006).

There have been efforts to create CpG-free plasmids that are specially engineered to completely eliminate CpG dinucleotides. Unlike standard plasmids, CpG-free plasmids do not stimulate the innate immune response. Indeed, Hyde et al. demonstrated that CpG-free plasmid DNA expression vectors do not provoke a robust immune response and do not activate TLR9 responses within the endosome. These plasmids also avoid DNA methylation in the nucleus, resulting in sustained transgene expression (Hyde et al., 2008). Additional studies have shown similar effects, including a longer duration of transgene expression and prevention of TLR9 activation and inflammation (Hyde et al., 2008; Reyes-Sandoval and Ertl, 2004; Dormiani et al., 2016; Habib et al., 2020).

If plasmids bypass TLR9, they may still undergo *de novo* methylation in the nucleus, leading to transgene silencing, (Alhaji et al., 2019) especially if they contain CpG-rich islands near the promoter (Tate and Bird, 1993). Godecke et al. examined DNA methylation as a mechanism for the silencing of an integrated lentiviral transgene using the endogenous promoters ROSA26 and ThumpD3 as controls. Bisulfite sequencing and EpiTYER analysis showed extensive methylation (e.g., methylation of >95% CpG motifs) of a bidirectional Tet promoter, which led to transgene silencing *in vitro* and *in vivo*. In contrast, endogenous reference promoters (ROSA26 and ThumpD3) remained largely unmethylated. This problem inspired Godecke et al. to develop an rtTA-TET1 fusion protein that could specifically bind and demethylate target genes with an upstream tetracycline-responsive element (TRE). Specifically, Ten-Eleven Translocation (TET) enzymes catalyze the stepwise oxidation of 5mC to 5-hydroxymethylcytosine (5hmC), which is converted to 5-formylcytosine (5fC) and 5-carboxycytosine (5caC). These oxidized intermediates are recognized and removed by the BER pathway, ultimately restoring unmethylated cytosines (Chen and Riggs, 2011). Consequently, induction of the rtTA-TET1 fusion protein with doxycycline led to a decrease in promoter methylation from 90% to 20% and a reactivation of the downstream reporter genes (luciferase and GFP)(Gödecke et al., 2017).

To test whether DNA methylation limits transient transgene expression from episomal plasmids, Hong et al. transfected C3A human hepatoblastoma cells with a GFP-encoding plasmid using Lipofectamine. GFP expression driven by the CMV promoter was measured after treating the cells with the DNMT inhibitor 5-azacytidine (5-aza) (Hong et al., 2001). To prevent plasmid loss during the experiment, the cells were growth-arrested for one week. Flow cytometry analysis revealed a dose-dependent increase in GFP expression with the highest 5-aza dose yielding a 150% increase in fluorescence and a higher proportion of GFP-positive cells. Methyl-Assisted PCR (MAP) was then used to show that a subset of CpG sites within the transgene cassette were unmethylated (Hong et al., 2001).

Alternatively, Jia et al. prevented *de novo* DNA methylation by using CRISPR-Cas9 to knock out DNMT3a in CHO cells. Specifically, they constructed episomal expression vectors with either a CMV or EF1α promoter driving GFP and transfected them into knockout and wildtype cells. The cells were then analyzed at regular intervals using flow cytometry, which demonstrated that the DNMT3a knockout reduced promoter methylation and enhanced long-term stability of CMV-driven transgene expression (Jia et al., 2018). Similarly, Robert et al. demonstrated that the knockdown of DNMT1 using antisense inhibitors and siRNAs resulted in decreased methylation in the endogenous tumor-suppressor gene and successfully reactivated the silenced tumor suppressor gene CDKN2A in HCT116 cells (Robert et al., 2003). These studies demonstrate that disrupting the function of DNMTs by employing knockout or knockdown methods reduces DNA methylation in plasmid DNA, thereby preventing methylation-mediated silencing of transgenes. Altogether, these findings indicate that DNA methylation can significantly suppress transgene expression from episomal plasmids, but inhibition of DNA methylation can rescue transgene expression.

However, it is important to reiterate that DNA methylation is not the only repressive mechanism that acts on transgenes. Indeed, Chen et al. showed that removing CpG motifs from plasmid DNA did not prevent silencing in murine liver tissue (Chen et al., 2008). Similarly, Zang et al. observed a negligible impact on the expression levels of transgenes containing CpG motifs in the liver after treating mice with the DNMT inhibitor 5-aza (Zang et al., 2015). Both studies concluded that methylation of CpG sites in plasmid DNA is not the only factor responsible for reduced transgene expression(Hyde et al., 2008). Therefore, future gene therapies should be designed to address multiple independent mechanisms of transgene expression, including DNA methylation, histone modifications, and others.

These sections collectively emphasize that transgene expression is influenced by multiple, interrelated epigenetic mechanisms in eukaryotes rather than a single regulatory pathway. Generally, histone acetylation promotes an open chromatin state and facilitates transcription, whereas histone methylation can either repress or activate gene expression, depending on the specific residue and modification pattern. Additionally, histones are valuable as gene delivery vehicles due to their positive charge, which enhances DNA binding and condensation, thereby improving plasmid packaging and cellular uptake. However, efficient delivery does not necessarily ensure sustained expression, as the introduced DNA remains susceptible to nuclear epigenetic silencing, particularly through CpG methylation. Therefore, effective transgene design must consider chromatin remodeling, DNA methylation, and delivery efficiency simultaneously to achieve lasting expression.

## 5. Conclusion

Several host cell defense mechanisms in the cytosol and nucleus act as barriers to efficient transgene delivery and expression. In the nucleus, transfected plasmids are detected by DNA sensors like IFI16. This leads to the assembly of a repressive complex known as the restrictosome, which initiates a cascade of epigenetic silencing events. Upon binding to foreign DNA, the restrictosome acts as a scaffold for the recruitment of additional effector proteins, including chromatin remodelers and histone-modifying enzymes such as histone deacetylases (HDACs), histone methyltransferases (HMTs) and DNA methyltransferases (DNMTs). These enzymes introduce repressive epigenetic marks. Histone deacetylation removes activating acetyl groups, while histone methylation establishes a compact chromatin structure that is transcriptionally silent, especially at lysine residues such as H3K9me3 and H3K27me3. Alternatively, DNA methylation at CpG islands further represses gene expression by recruiting methyl-CpG-binding domain proteins (MBDs). These proteins attract additional silencing complexes, including HP1 and polycomb group proteins, which stabilize heterochromatin and lead to long-term gene silencing. These multilayered and redundant mechanisms are robust antiviral defense strategies, but they also pose a significant challenge for gene therapy. Overall, this review highlights the necessity for advanced vector and transgene engineering approaches that can bypass or prevent innate immune responses, resist epigenetic silencing, and enhance chromatin accessibility to achieve long-lasting transgene expression to improve current and future gene therapies.

## Figures and Tables

**Fig. 3.**
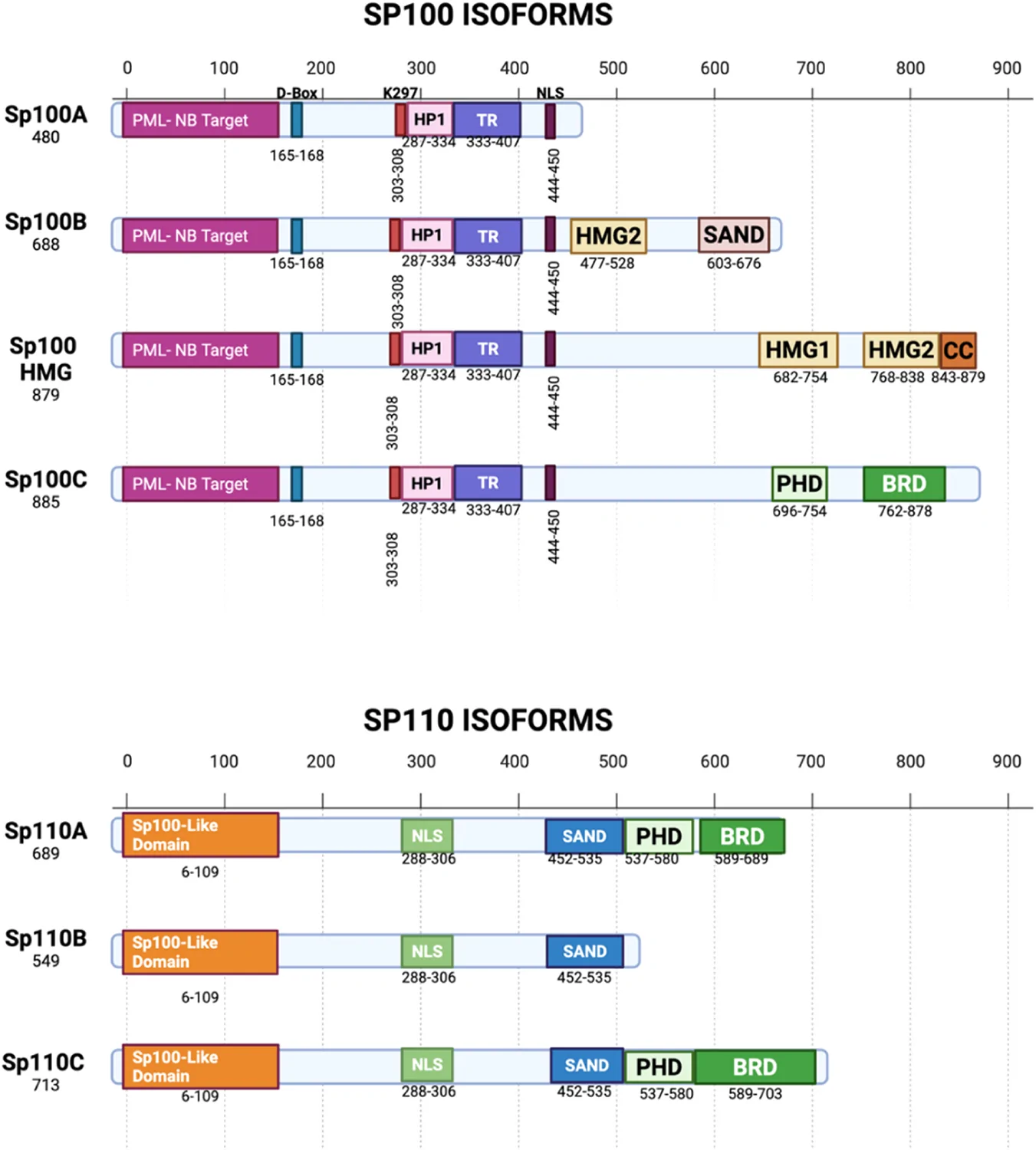
Composition of Sp100 and Sp110 splice isoforms (Collados Rodríguez, 2020; Lehming et al., 1998; Negorev et al., 2006). Sp100 isoforms (Sp100A/B/C/HMG) share the N-terminal 477 amino acids (aa) containing: dimerization and PML-NB targeting (Amino Acid(aa) 3–152, pink), destruction-box (D-box, aa 165–168, teal); HP1 interacting region (aa 287–334, light pink) with Lysine 297 SUMO modification (K297, red and SUMO-interacting motif (SIM, aa 323–326); trans-activating region (TR, aa 333–407, purple), autoepitopes, and nuclear localization sequence (NLS, aa 440–450, burgundy). Sp100B/C/HMG are identical up to aa 685, including a high mobility group 2 (HMG2, aa 477–528, dark yellow) and SAND DNA-binding domain (aa 603–676, beige). Sp100HMG adds HMG1 (aa 682–754, yellow), HMG2 (aa 768–838), and coiled coil (CC, aa 843–879, orange). Sp100C includes a plant homeodomain (PHD, aa 696–754, light green) and a bromodomain (BRD, aa 762–878, green). All Sp110 isoforms have an Sp100-like domain (aa 6–109, orange), a nuclear localization sequence (NLS, aa 288–306, green), and a SAND domain (aa 452–535, dark blue). Sp110A and Sp110 C have a PHD Domain (aa 537–580, light green). Sp110 A has a BRD domain (aa 589–679, dark green) and Sp110 C has a BRD domain (aa 589–703). This figure was created in BioRender.

**Table 1:** Nuclear restriction factors relevant to plasmids.

Pathway	Proteins	Effect on Plasmids	Function	Ref.
Cytosolic DNA	AIM2	Promotes heterochromatinization of foreign	Binds bare foreign DNA, oligomerizes through pyrin domain, recruits nuclear bodies and silences transgenes.	^(^Merkl et al., 2018; Orzalli et al., 2013; Roy et al., 2019)
Sensors	IFI16	DNA by recruiting innate immune response proteins.		
	cGAS			
Epigenetic	SMC5/6	Represses transcription and silence DNA and transgene expression	Interacts with SIMC1-SLF2 and promotes a repressive chromatin state.	^(^Oravcová et al., 2025; Roubille et al., 2024)
Silencing	Complex			
	HUSH		Recruits SETDB1 and MORC2 which are effectors that recruit H3K9me3 leading to transcriptional repression	
ND10/PML	PML	Represses transcription and silence DNA and transgene expression	Deposits repressive chromatin marks, H3.3 and H3K9me3, repressing and silencing transgenes	^(^Merkl et al., 2018; Merkl and Knipe, 2019; Cabral et al., 2018; Ishov et al., 1999; He et al., 2015; Elsässer et al., 2012)
Nuclear Body	DAXX			
Pathway	ATRX			

**Table 2:** Summary of restrictosome inhibition/knockout studies.

Target(s)	Cell Type	Transgene Promoter	Vehicle	Effects of Knockouts	Ref.
IFI16	HFF	N/A	HSV–1	Increased expression of viral proteins, increased viral replication	^(^Merkl et al., 2018; Sutter et al., 2024; Johnson et al., 2014)
	U2OS	CMV	rAAV2		
	MCF–7	N/A			
PML	HFF	N/A	HSV–1	Increased viral replication, decreased viral immediate early and early transcript levels, increased viral vector transduction	^(^Merkl et al., 2018; Ma et al., 2022; Mitchell et al., 2014)
	HeLa	CMV	rAAV1		
	TE671	N/A			
ATRX	U87T	N/A	LV	Loss of chromatin accessibility, loss of heterochromatin, prevented localization of DAXX to nuclear foci	^(^He et al., 2015; Gulve et al., 2022; Clatterbuck Soper et al., 2024)
	G292	CMV			
	mESCs	N/A			
DAXX	HFF	N/A	HSV–1	Increased viral replication and viral expression	^(^Merkl et al., 2018; Xu et al., 2016)
	Hep2	CMV			
Sp100B	Hep–2	CMV	HSV–1	Inhibition of viral infection, decreased viral and reporter transgene expression, reduced chromatin silencing	^(^Ma et al., 2022; Xu et al., 2016; Liu et al., 2018)
Sp100C	HEK–293	N/A			
Sp100					
HMG					

**Table 3:** Histone modifications observed on plasmid nucleosomes.

Histones	Residues	Modifications	Role
H2A	K119	Monoubiquitinylation (ub)	Transcriptional Repression (Zhang et al., 2017)
H2B	K120	Ubiquitinylation (ub)	Transcriptional Activation (Zhu et al., 2005)
H3	K4	Trimethylation (me3)	Active Euchromatin (Kouzarides, 2007)
H3	K9	Trimethylation (me3)	Transcriptional Repression (Kouzarides, 2007)
H3	K27	Trimethylation (me3)	Transgene Silencing (Kouzarides, 2007)
H3	K4, K9	Acetylation (ac)	Transcriptional Activation (Kouzarides, 2007)
H4	K16	Acetylation	Transcriptional activation (Shogren-Knaak et al., 2006)

## Data Availability

No data was used for the research described in the article.

## Abbreviations

SUMO: Small Ubiquitin-like Modifier
ISGs: Interferon IFN-Stimulated Genes
HSV: Human Simplex Virus
ICP0: Infected Cell Protein 0
IE1: Immediate Early Protein 1
IFI16: Interferon Gamma Inducible Protein 16
PML: Promyelocytic Leukemia
ATRX: Alpha- thalassemia/mental retardation, X-linked
DAXX: Death Domain-Associated Protein 6
